# Hepatitis B Virus Lacks Immune Activating Capacity, but Actively Inhibits Plasmacytoid Dendritic Cell Function

**DOI:** 10.1371/journal.pone.0015324

**Published:** 2011-01-05

**Authors:** Andrea M. Woltman, Marjoleine L. Op den Brouw, Paula J. Biesta, Cui C. Shi, Harry L. A. Janssen

**Affiliations:** 1 Department of Gastroenterology and Hepatology, Erasmus MC - University Medical Center, Rotterdam, The Netherlands; 2 Department of Infectious Disease, Rujin Hospital, Shanghai, China; Hannover Medical School, Germany

## Abstract

Chronic hepatitis B virus (HBV) infection is caused by inadequate anti-viral immunity. Activation of plasmacytoid dendritic cells (pDC) leading to IFNα production is important for effective anti-viral immunity. Hepatitis B virus (HBV) infection lacks IFNα induction in animal models and patients and chronic HBV patients display impaired IFNα production by pDC. Therefore, HBV and HBV-derived proteins were examined for their effect on human pDC *in vitro*. In addition, the *in vitro* findings were compared to the function of pDC derived from chronic HBV patients *ex vivo*. In contrast to other viruses, HBV did not activate pDC. Moreover, HBV and HBsAg abrogated CpG-A/TLR9-induced, but not Loxoribine/TLR7-induced, mTOR-mediated S6 phosphorylation, subsequent IRF7 phosphorylation and IFNα gene transcription. HBV/HBsAg also diminished upregulation of co-stimulatory molecules, production of TNFα, IP-10 and IL-6 and pDC-induced NK cell function, whereas TLR7-induced pDC function was hardly affected. In line, HBsAg preferentially bound to TLR9-triggered pDC demonstrating that once pDC are able to bind HBV/HBsAg, the virus exerts its immune regulatory effect. HBV not only directly interfered with pDC function, but also indirectly by interfering with monocyte-pDC interaction. Also HBeAg diminished pDC function to a certain extent, but via another unknown mechanism. Interestingly, patients with HBeAg-positive chronic hepatitis B displayed impaired CpG-induced IFNα production by pDC without significant alterations in Loxoribine-induced pDC function compared to HBeAg-negative patients and healthy controls. The lack of activation and the active inhibition of pDC by HBV may both contribute to HBV persistence. The finding that the interaction between pDC and HBV may change upon activation may aid in the identification of a scavenging receptor supporting immunosuppressive effects of HBV and also in the design of novel treatment strategies for chronic HBV.

## Introduction

Hepatitis B virus (HBV) infects the liver as primary target and may elicit progressive liver injury leading to increased risk of developing liver cirrhosis, liver failure and liver cancer [1]. Chronic infection with HBV is the result of an ineffective anti-viral immune response towards the virus [1]–[3]. The exact mechanism by which HBV escapes immunity is still not known.

In general, the immune system is alerted and evokes a number of mechanisms that are aimed at eradicating the viral attack immediately following viral infection. The initial response to viral infection is the rapid release of type I interferons (IFN), IFNα and IFNβ, which is observed for most viruses studied [4]. These IFN enhance the first defense against viral infections and modulate both innate and adaptive immune cells. Indications of the role of type I IFN during HBV infection are mostly based on studies in chimpanzees, since this is the only animal that can be infected with HBV. In sharp contrast to other viruses including hepatitis C virus, chimpanzees infected with HBV showed a complete lack in the induction of type I IFN and in IFN-response genes during the early stages of infection [5]. It is difficult to study the early events of acute HBV infection in humans. Nevertheless, it was recently shown that type I IFN responses are also lacking in acute HBV patients [6].

Plasmacytoid dendritic cells (pDC) are the principal producers of type I IFN and play a central role in immune responses against viral infections [7], [8]. pDC respond to viruses and other pathogens primarily through the recognition of pathogen-associated molecular patterns by two intracellular Toll-like receptors (TLR), TLR7 and TLR9, which recognizes single stranded RNA and unmethylated DNA motifs, respectively [9], [10]. TLR-triggering activates pDC to rapidly produce high levels of type I interferons, but also other cytokines, including TNF-α and IL-6, and cell surface co-stimulatory molecules. In this way pDC exert a direct anti-viral effect by producing factors that inhibit viral replication, but they also activate natural killer (NK) cells and T lymphocytes allowing further priming and regulation of anti-viral immunity [7], [11], [12].

Circulating blood pDC numbers seem to be unaffected by HBV, but functional deficits in pDC from chronic HBV patients including impaired IFN-α production have been reported [13]. Recently, it was reported that patient-derived HBsAg binds to human pDC *in vitro* and impairs TLR9-induced IFNα production by pDC [14]. The presence of HBV-DNA in or on pDC *in vivo* in chronic HBV patients [15], [16] indicates that at least the whole virus and not only HBsAg interacts with pDC. Whether also HBV particles and/or other HBV-derived proteins present in patient's circulation interfere with pDC function is not known. Given the central role that pDC play in antiviral immune responses, understanding the mechanisms whereby pDC interact with and respond to HBV may provide fundamental insights into the regulation of HBV-specific immunity and the development of HBV chronicity. Therefore, the present study investigated the effect of HBV as whole particles and HBV-derived proteins, i.e. HBcAg, HBeAg and HBsAg, on direct and indirect anti-viral functions of pDC.

## Results

### HBV does not activate pDC

**Figure 1:**
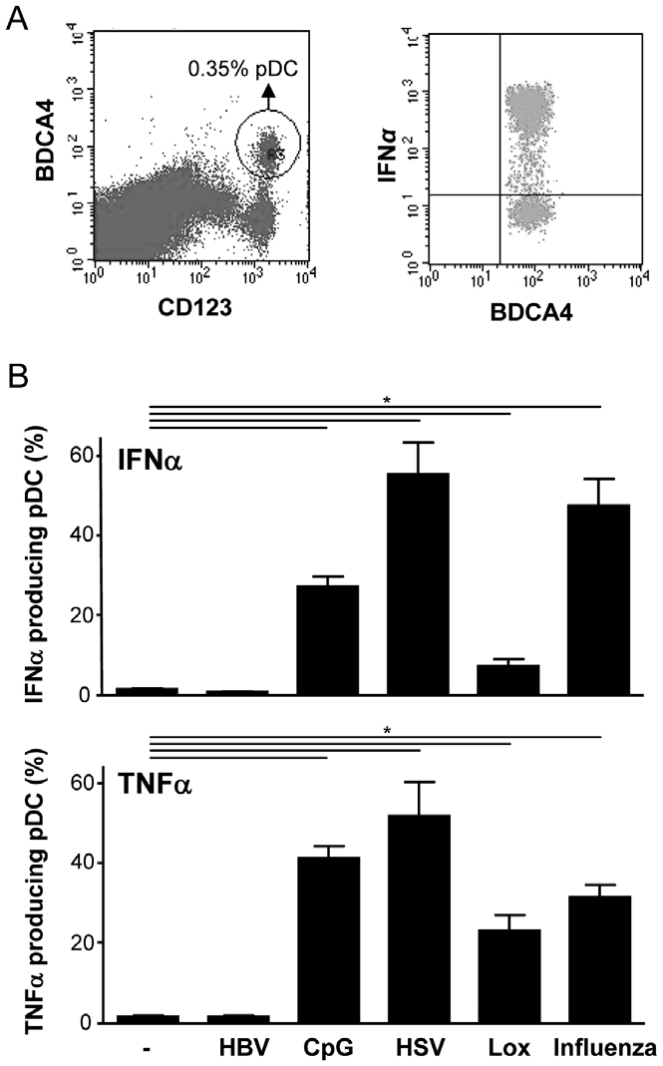
HBV does not activate pDC. **A**: PBMC were stimulated with CpG and analysed for IFNα production by pDC as described in *Materials and Methods*. Shown is a representative FACS plot of PBMC stained for CD123 and BDCA-4 to identify pDC and the detection of IFNα positive cells within this pDC-gate. **B**: IFNα and TNFα producing pDC within PBMC cultured in the presence or absence of HepG2.215-derived HBV, CpG, HSV-1, Lox or Influenza are presented as mean±SEM of 10 independent experiments.

HBV is a DNA virus that replicates via an RNA intermediate. In theory, HBV may thus be able to activate pDC via TLR7, TLR9 and/or cytosolic pattern recognition receptors. However, no evidence exists that HBV replicates in pDC, which makes the direct activation of pDC by HBV via TLR7 maybe not very likely. Known synthetic and viral TLR7 and TLR9 ligands including Influenza virus, HSV-1, CpG and to a lesser extent Lox induced pDC to produce IFNα (Fig. 1A). In contrast, HBV did not give rise to IFNα producing pDC (Fig. 1). Similar data were observed for TNFα.

Though HBV did not induce cytokines, the virus might induce pDC maturation. Whereas HSV-1 and Influenza virus as well as the synthetic TLR7 and TLR9 ligands upregulated the expression of CD40, CD80, CD86, and to a minor extent HLA-DR, HBV only marginally increased the expression of HLA-DR without affecting the expression of CD40, CD80 and CD86 (Table 1).

**Table 1:** Effect of HBV on pDC maturation.

	CD40		CD80		CD86		HLA-DR	
	ctr	HBV	ctr	HBV	ctr	HBV	ctr	HBV
**Medium**	6.8±1.7	5.0±0.1	23.3±9.4	17.4±6.0	46.4±21.1	34.5±15.6	791.2±385.1	993.4±396.6
**HBV**	5.0±0.1	nd	17.4±6.0	nd	34.5±15.6	nd	993.4±396.6	nd
**CpG**	107.1±15.1	32.2±5.3*	89.2±25.4	59.9±26.4	121.5±33.2	46.9±20.4*	911.2±256.8	1250.6±419.4
**HSV**	61.5±21.0	49.6±19.2*	37.7±12.8	41.7±19.2	71.8±27.0	84.7±41.7	836.9±206.1	1109.5±425.0
**Lox**	44.2±2.5	44.2±5.3	72.7±20.7	89.1±27.4	100.8±20.5	114.7±32.1	1136.1±225.9	1368.4±377.4
**Influenza**	153.5±23.5	142.2±19.1	90.2±9.7	89.1±11.1	169.2±25.2	137.3±9.7	779.8±158.3	855.9±174.4

To investigate possible pDC activation by HepG2.215-derived HBV in comparison with other pDC stimuli, PBMC were cultured in the presence or absence of HBV (100 geq/cell), CpG, HSV-1, Lox or Influenza (see ‘ctr’ lane). To investigate the immune regulatory effect of HBV on pDC maturation induced by other known pDC activating ligands, PBMC were cultured with or without CpG, HSV-1, Lox or Influenza in the presence (‘HBV’ lane) or absence (‘ctr’ lane) of HBV (100 geq/cell). After 24h, cells were harvested and the expression of CD40, CD80, CD86 and HLA-DR on pDC was determined by flow cytometry. Data are presented as mean±SEM fluorescent intensity of 3 independent experiments with different donors. Similar data were observed for purified pDC (not shown).

*p<0.05, paired t-test; nd = not done.

### HBV impairs CpG-induced pDC maturation and function

To determine whether HBV mainly behaves as a stealth virus for pDC, or that it has an active role in the regulation of pDC function. pDC were activated with HSV-1, Lox and Influenza virus either with or without HBV. HBV significantly inhibited HSV-induced CD40 expression. Most pronounced inhibitory effects of HBV were observed for CpG-induced pDC maturation as demonstrated by diminished CD40, CD80 and CD86 upregulation (Table 1). As was found for the minor increase in HLA-DR expression upon HBV in non-stimulated cultures, HBV slightly upregulated HLA-DR expression in stimulated pDC cultures, which was not significant for the individual stimuli, but was significantly elevated by HBV when combining all different stimuli.

**Figure 2:**
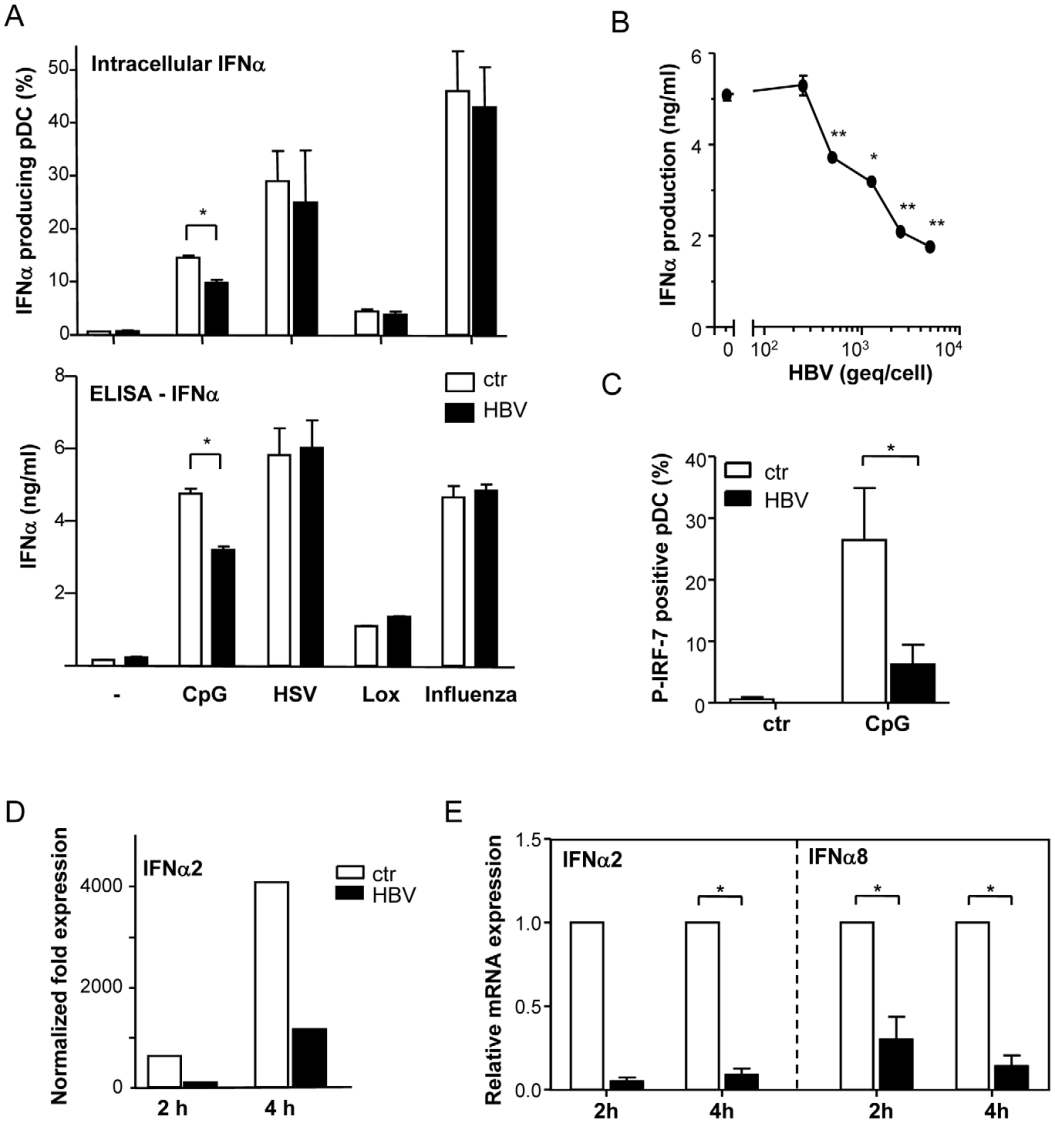
HBV dose-dependently inhibits CpG-induced transcription of IFNα in pDC. **A**: IFNα production by pDC cultured in medium, CpG, HSV-1, Lox or Influenza with or without HepG2.215-derived HBV was determined by flow cytometry or ELISA. Mean±SEM of 3 independent experiments. **B**: CpG-activated pDC were cultured with different doses of HepG2.215-derived HBV. IFNα production determined by ELISA is presented as mean±SEM of 16 independent experiments with different donors. **C**: pDC were cultured with CpG in the presence or absence of HepG2.215-derived HBV for 3h. Data present the mean±SEM percentage of cells positive for phosphorylated IRF7 from 5 independent experiments. **DE**: pDC were cultured with CpG in the presence or absence of HepG2.215-derived HBV for 2 or 4h. Data represent IFNα2 (**DE**) and IFNα8 (**E**) mRNA levels normalized to GAPDH and are representative for 6 experiments (**D**) or show the mean±SEM IFNα mRNA levels relative to cultures without HBV (n = 6) (**E**). *p<0.05, **p<0.01, p<0.001, Wilcoxon signed rank test.

HBV did not affect the IFNα production induced by these stimuli (Fig. 2A), except for CpG-induced IFNα, which was dose-dependently inhibited by HBV as assessed by intracellular flow cytometry after 5h (Fig. 2A) and ELISA after 24h (Fig. 2AB) and 48h (data not shown) of culture. Since HSV-1 can trigger TLR9, but also other innate immune receptors, and Lox and Influenza are known to trigger TLR7, the inhibitory effect of HBV seemed to be restricted to TLR9 triggering.

To assess whether HBV interferes with IFNα production at the transcriptional level, pDC were stimulated with CpG in the presence or absence of HBV, and analysed by intracellular flow cytometry for the presence of phosphorylated IRF7, the transcription factor involved in the transcription of IFNα genes. HBV reduced CpG-induced IRF7 phosphorylation (Fig. 2C) and in line also reduced IFNα2 and IFNα8 mRNA levels as detected by quantitative RT-PCR analysis (Fig. 2DE).

### HBV inhibits cytokine production and NK cell activation by pDC

**Figure 3:**
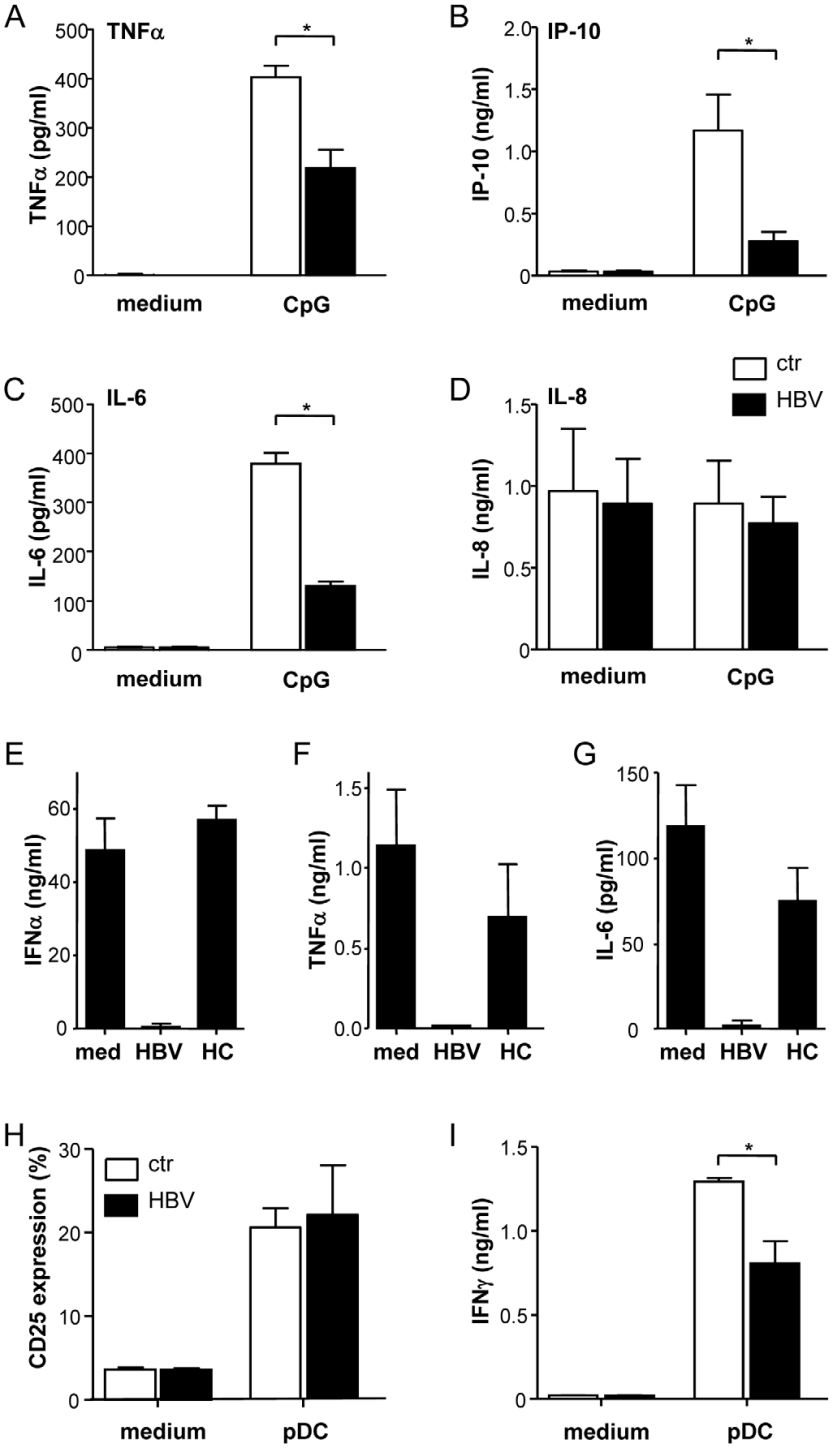
HBV inhibits cytokine production and pDC-induced NK cell activation. **ABCD**: Purified pDC were cultured with CpG in the presence or absence HepG2.215-derived HBV. Supernatants were analysed for TNFα (A), IP-10 (B), IL-6 (C) and IL-8 (D). Data demonstrate mean±SEM of 8 independent experiments. **EFG**: Purified pDC were stimulated with CpG in the presence or absence of patient serum-derived HBV. Healthy control serum was treated in a similar way and added in the same volume to pDC. After 24h, supernatants were harvested and tested for the presence of IFNα (E), TNFα (F) and IL-6 (G) by ELISA. Shown is the mean±SD of triplicate cultures from one out of 2 experiments with different donors. **HI**: NK cells were cultured with or without pDC and with or without HepG2.215-derived HBV in medium containing IL-3 and CpG. Data show mean±SEM CD25 expression on CD56+ cells (E) and IFNγ production (F) of 7 independent experiments. *p<0.05, Wilcoxon signed rank test.

Next to IFNα, pDC produce more cytokines involved in anti-viral immunity. According to previous studies, CpG stimulation resulted in the induction of TNFα, IP-10 and IL-6 (Fig. 3A–C), but not IL-8 (Fig. 3D). HBV inhibited TNFα, IP-10 and IL-6 production, whereas IL-8 production was not significantly affected (Fig. 3A–D). In line with this cell-culture derived HBV, also patient-derived HBV particles inhibited CpG-induced production of IFNα (3E), TNFα (F) and IL-6 (G) by pDC.

Besides the direct antiviral effects of the cytokines, pDC also have indirect anti-viral activities via the activation NK cells [12]. Addition of CpG-activated pDC to NK cells resulted in strong NK cell activation as demonstrated by the upregulation of CD69, CD25 and the induction of IFNγ production (Fig. 3HI; data not shown). Although HBV did not affect pDC-induced upregulation of CD25 on NK cells (Fig. 3H), NK cell-derived IFNγ production induced by pDC was significantly decreased (Fig. 3I). Supernatants derived from non-transfected HepG2 cells served as a control and did not influence pDC function (data not shown).

### Monocytes support immune regulatory effect of HBV on pDC

**Figure 4:**
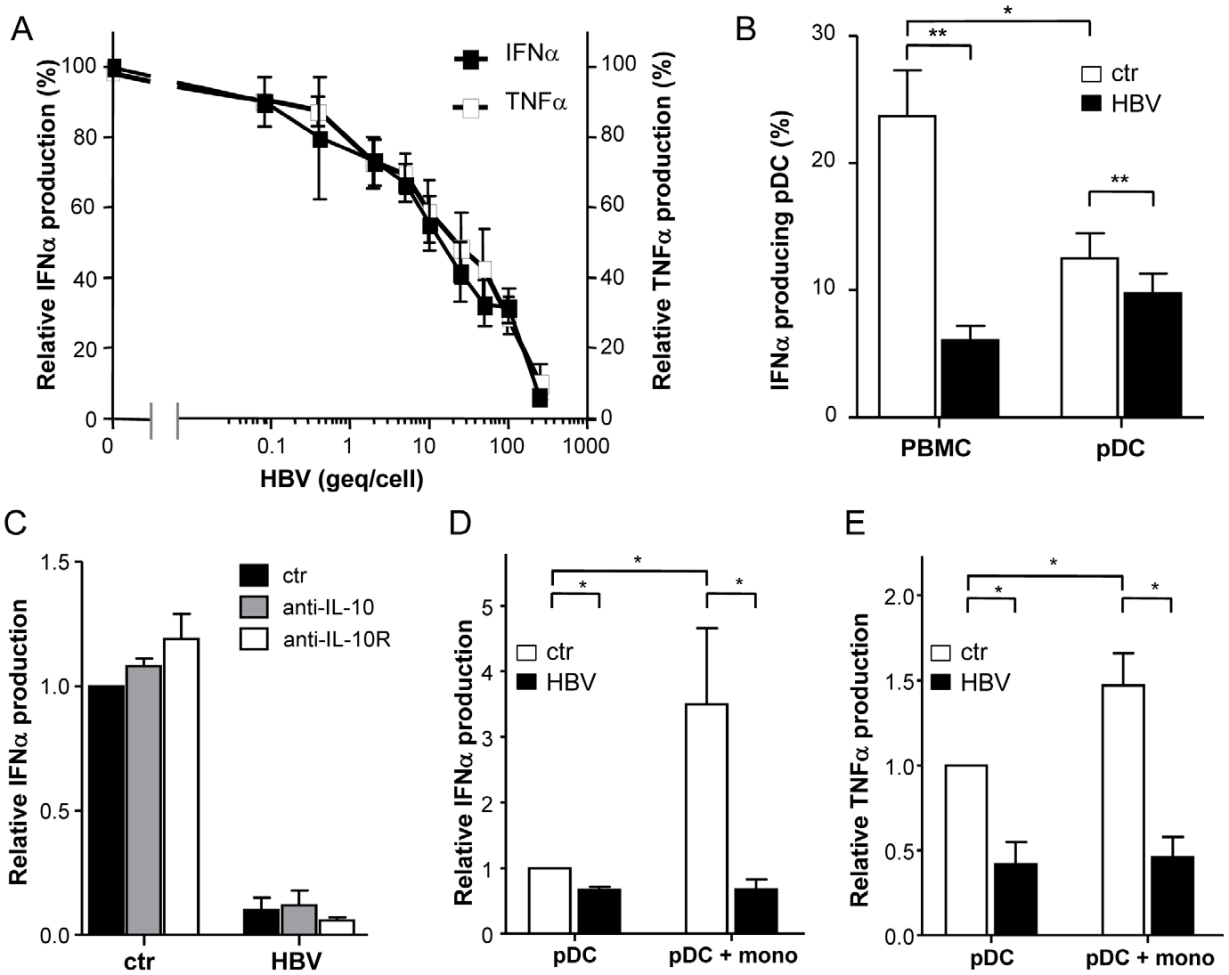
Monocytes support immune regulatory effect HBV on pDC. **A**: PBMC were stimulated with CpG with or without HepG2.215-derived HBV. Data present mean±SEM frequencies of IFNα and TNFα producing pDC of 6 independent experiments. **B**: PBMC or pDC were cultured with CpG with or without HepG2.215-derived HBV. Mean±SEM intracellular IFNα expression in pDC of 21 independent experiments is shown. *p = 0.01, **p<0.001, Wilcoxon signed rank test. **C**: PBMC were stimulated with CpG with or without HepG2.215-derived HBV and with or without neutralizing antibodies to IL-10 or IL-10R. The percentages of IFNα producing pDC were determined by flow cytometry. The percentage of cytokine producing pDC in control cultures was set on 1 and the relative production was calculated. Data demonstrate mean±SEM relative IFNα production by pDC (n = 3). **DE**: pDC were cultured alone or with monocytes (1∶5) and stimulated with CpG either in the presence or absence of HepG2.215-derived HBV (1000 geq/pDC). The percentage of IFNα (**D**) and TNFα (**E**) producing pDC were determined by flow cytometry. Due to large variation between donors, the percentage of cytokine producing pDC in control pDC cultures was set on 1 and the relative cytokine production was calculated. Wilcoxon signed rank tests were performed on original data, *P<0.05 (n = 6).

Although on a per cell basis more HBV particles seemed to be required for significant immune regulatory effects on purified pDC compared to pDC present in PBMC cultures, the number of HBV genome equivalents per pDC in pure pDC cultures compared to the ±0.3% pDC present in total PBMC cultures was even 10–50 times less (Fig. 2B, 4A). Nevertheless, the relative inhibition of CpG-induced IFNα producing pDC was more pronounced when whole PBMC cultures were exposed to HBV (Fig. 4B). This was not simply due to positive selection on BDCA4 as suggested before [17], since negative pDC selection revealed similar results (data not shown). A role for IL-10 produced by other cell types as an explanation for the stronger inhibition found in total PBMC cultures could also be excluded since neutralizing IL-10 or its receptor neither changed IFNα production nor TNFα production by pDC (Fig. 4C; data not shown).

To identify the cell type responsible for the enhanced IFNα production by pDC in total PBMC cultures, purified pDC were compared with purified pDC supplemented with CD3^+^, CD14^+^, CD19^+^, or CD56^+^ cells. Only addition of monocytes to pDC enhanced the frequency of IFNα-producing and pDC (Fig. 4D; data not shown). This extra monocyte-mediated IFNα production by pDC was completely abolished by HBV (Fig. 4D). Monocytes also enhanced the frequency of TNFα producing pDC upon stimulation with CpG, but to a lesser extent compared to IFNα (Fig. 4E). This resulted in a significantly enhanced inhibitory effect of HBV on IFNα-producing pDC in the presence of monocytes (33.4±4.8% inhibition by HBV for pDC *vs* 64.6±12.2% for pDC+mono), but a relatively unaltered rate of inhibition of TNFα-producing pDC in the presence of monocytes (58.2±12.9% for pDC, 63.1±11.0% HBV-induced inhibition for pDC+mono). These data indicate that the supporting function of monocytes regarding TLR9-induced cytokine production, especially IFNα, is abrogated by HBV.

### HBeAg and HBsAg, but not HBcAg diminish pDC function

**Figure 5:**
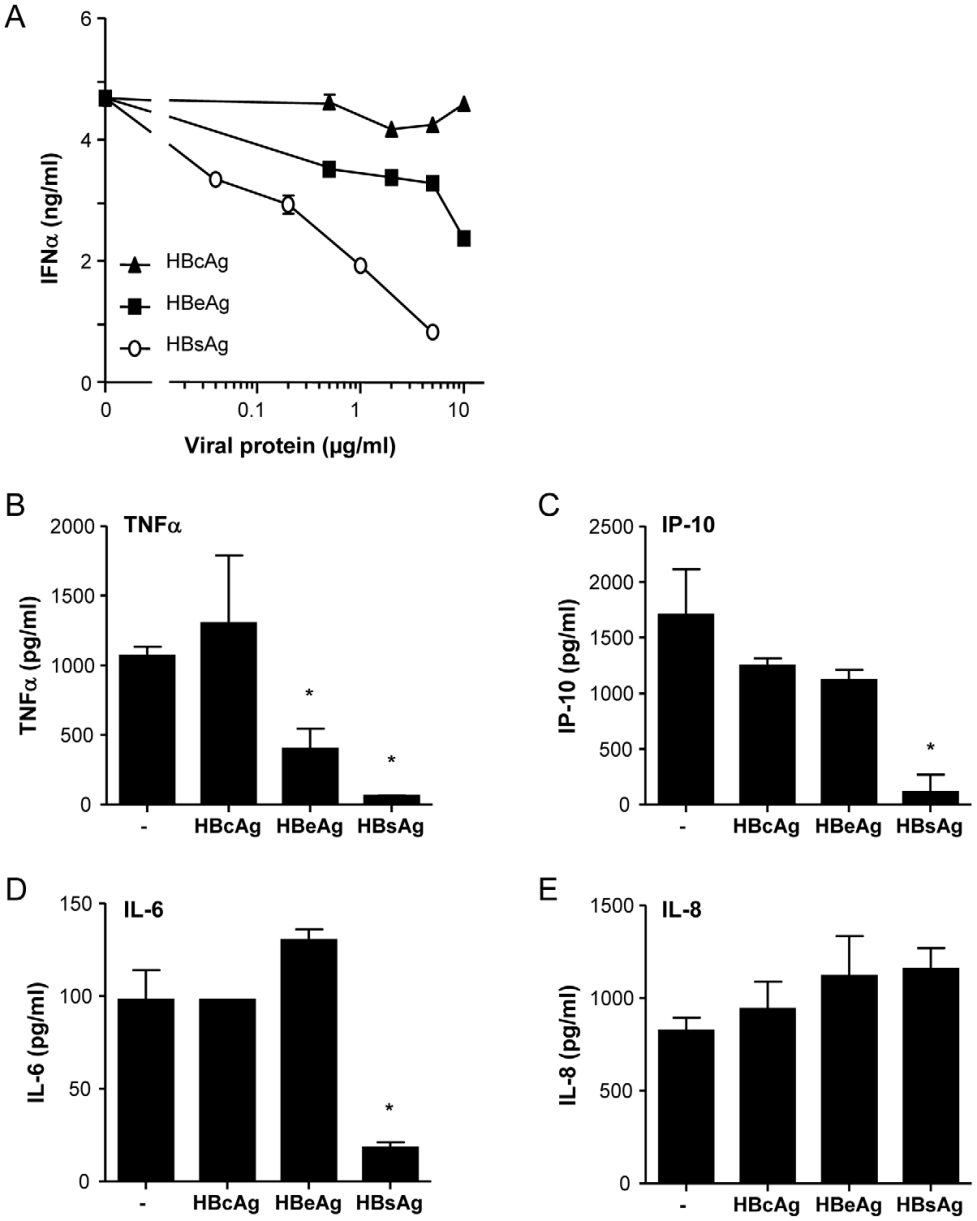
HBsAg and HBeAg inhibit cytokine production by pDC. pDC were cultured with CpG in the presence or absence of increasing doses or 5 µg/ml of HBcAg, HBeAg or HBsAg. Supernatants were harvested and analysed for IFNα (A), TNFα (B), IP-10 (C), IL-6 (D), and IL-8 (E). Data presented are mean±SEM of at least 8 independent experiments. *P<0.05, Wilcoxon signed rank test compared to control.

Next to whole virus also HBV-derived proteins present in patients' circulation, including HBeAg and HBsAg, may affect pDC function [18]. Therefore HBsAg, HBcAg, and HBeAg were investigated for their effect on IFNα production. Exposure of pDC to the viral proteins alone, did not induce IFNα production (data not shown). Interestingly, dose response studies revealed that HBeAg and especially HBsAg, but not HBcAg dose-dependently reduced CpG-induced IFNα production by pDC (Fig. 5A).

Similar to the effect of whole virus, HBsAg inhibited CpG-induced TNFα, IP-10 and IL-6 production without significantly altering the production of IL-8 (Fig. 5B–E). HBeAg also reduced TNFα production, but did not significantly influence the secretion of the other cytokines. HBcAg did not modulate pDC function (Fig. 5). Of note, neither HBV nor its viral proteins reduced pDC viability as determined by flow cytometric analysis of intracellular active caspase-3 as well as the binding of Annexin-V and 7AAD (data not shown). In addition, experiments performed in the presence of polymyxin B to neutralize possible non-detectable levels of contaminating endotoxins did not affect the regulatory effects of HBV or the viral proteins (data not shown).

### HBeAg positive chronic HBV is associated with impaired CpG-induced pDC function

**Figure 6:**
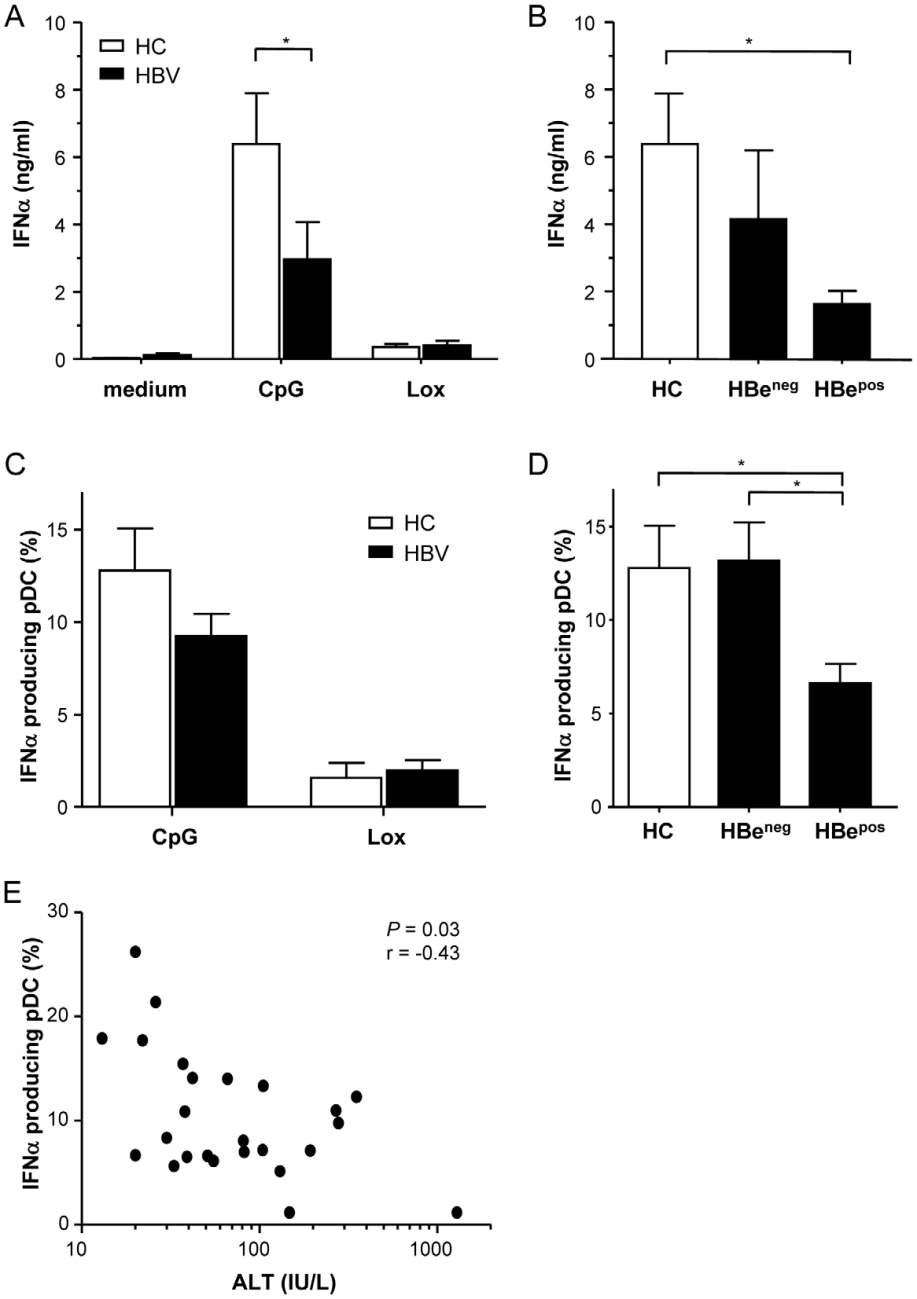
Chronic HBV patients display impaired CpG-induced pDC function. Frozen PBMC from patients (HBV; n = 25) and healthy controls (HC; n = 21) were thawed, washed and cultured in the absence or presence of CpG or Lox and investigated for the production of IFNα by ELISA (AB) or intracellular flow cytometry (CDE) as described in *Materials and Methods*. For additional analysis of the CpG-induced IFNα producing capacity, the patient group was divided into HBeAg-positive (n = 15) and HBeAg-negative (n = 10) disease. Shown are the mean±SEM IFNα production by HC and patients (A–D) and the relation between serum ALT levels and IFNα producing pDC within the total patient group as defined by Spearman's correlation coefficient. *Mann-Whitney U test, *P*<0.05.

To investigate whether pDC circulating in chronic HBV present a similar functional profile as observed after in vitro exposure to HBV or its viral proteins, CpG and Lox-induced IFNα production by pDC of chronic HBV patients was compared to the IFNα production by pDC of age and gender-matched healthy controls (Table 2). Impaired IFNα production by pDC from chronic HBV patients have been described before (reviewed in Woltman et al. [13]), but a comparison between TLR7 and TLR9 stimulation is lacking. Both CpG and Lox-stimulation significantly induced IFNα production as measured by ELISA and intracellular flow cytometry (Fig. 6A–D), albeit for Lox to a much lesser extent. Interestingly, only the CpG-induced IFNα production was significantly reduced in HBV patients compared to healthy controls, as was found for the *in vitro* effects of HBV. Additionally, when comparing HBeAg positive with HBeAg negative patients, we found that especially HBeAg-positive patients displayed a defect in the CpG-induced IFNα production (Fig. 6BD). This more pronounced impaired IFNα producing capacity of pDC from HBeAg positive patients may reflect the immunosuppressive effect of HBeAg, as demonstrated in the *in vitro* experiments, but may also be a more indirect effect of the significant higher viral load and/or ALT levels in HBeAg-positive patients (Table 2). When investigating a possible relation between the IFNα producing capacity and viral load or ALT, we found that serum HBV-DNA levels did not correlate to IFNα production (data not shown), whereas serum ALT levels significantly correlated with IFNα (Fig. 6E).

**Table 2:** Patient characteristics.

	N	Sex (%)	Age (yr)	HBV-DNA (Log_10_ IU/mL)	ALT (IU/L)
		F/M	Mean±SEM	Mean (Range)	Mean±SEM
**Healthy controls**	21	48/52	33.5±1.7	n.a.	n.d.
**Chronic HBV**	25	44/56	33.5±2.1	6.8 (≤3.0–10.4)	141±51
HBeAg^pos^	15	47/53	31.6±3.0	7.9 (≤3.0–10.4)	188±81
HBeAg^neg^	10	40/60	36.5±2.6	5.2 (≤3.0–9.8)	70±32*

n.a., not applicable; n.d., not determined;

*HBeAg^pos^ *vs* HBeAg^neg^, *P*<0.05.

### HBV/HBsAg diminishes S6 phosphorylation

**Figure 7:**
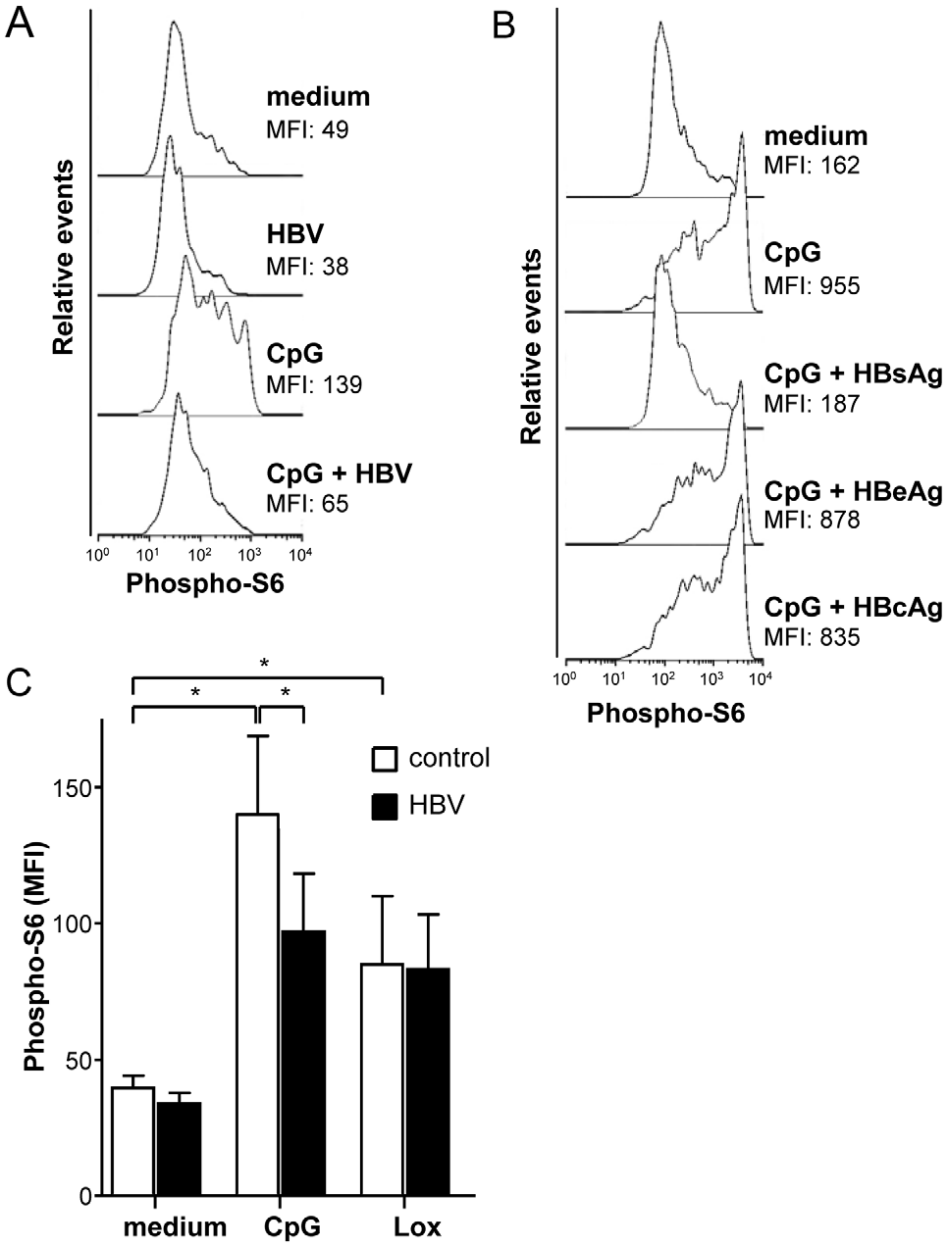
HBV and HBsAg inhibit CpG-induced S6 phosphorylation. **A**: pDC were cultured with CpG in the presence or absence of HepG2.215-derived HBV. Data show the expression of phosphorylated S6 and the mean fluorescence intensity (MFI) and are representative for 8 independent experiments. **B**: pDC were cultured with or without CpG in the presence or absence of HBcAg, HBeAg, or HBsAg. Data show expression of phosphorylated S6 and the MFI and are representative for 3 independent experiments. **C**: Data show mean±SEM expression of phosphorylated S6 in BDCA4^+^CD123^+^ pDC in PBMC exposed to CpG or Lox in the presence or absence of HepG2.215-derived HBV from 6 independent experiments. *p<0.05, Wilcoxon signed rank test.

To investigate the specific interference of HBV with CpG-induced pDC function in more detail, intracellular TLR7 and TLR9 signalling was examined. It was recently found that TLR/Myeloid differentiation primary response protein 88 (MyD88)-induced IFNα production by pDC requires mTOR activation [19] resulting in phosphorylation of IRF7 and subsequent IFNα gene transcription. To assess whether HBV interferes with mammalian target of rapamycin (mTOR)-induced IRF7 phosphorylation, pDC were stimulated with or without CpG or Lox in the presence or absence of HBV, and analysed for the expression of phosphorylated S6, a downstream target of mTOR [20]. Baseline S6 phosphorylation was low and hardly affected by HBV (Fig. 7A), HBsAg and HBcAg. Only HBeAg slightly increased S6 phosphorylation to an almost negligible extent (data not shown). As expected, CpG strongly increased S6 phosphorylation (Fig. 7A), which was significantly inhibited by HBV and HBsAg, but not by HBcAg or HBeAg (Fig. 7AB). Also TLR7 triggering induced phosphorylation of S6, but in contrast to TLR9 stimulation, neither HBV nor HBsAg significantly affected Lox-induced S6 phosphorylation (Fig. 7C).

### TLR9 triggering increases pDC-HBsAg interaction

Of course, the lack of inhibitory effects of HBV and HBsAg on Lox-induced S6 phosphorylation is completely in line with the absence of immune regulatory effects of HBV and HBsAg on Lox-induced pDC function. However, in case HBV actively induces a regulatory signalling molecule able to inhibit TLR9 signalling, e.g. SOCS proteins, these inhibitory proteins often block both TLR7 and TLR9 signalling, since these signalling pathways are quite similar.

**Figure 8:**
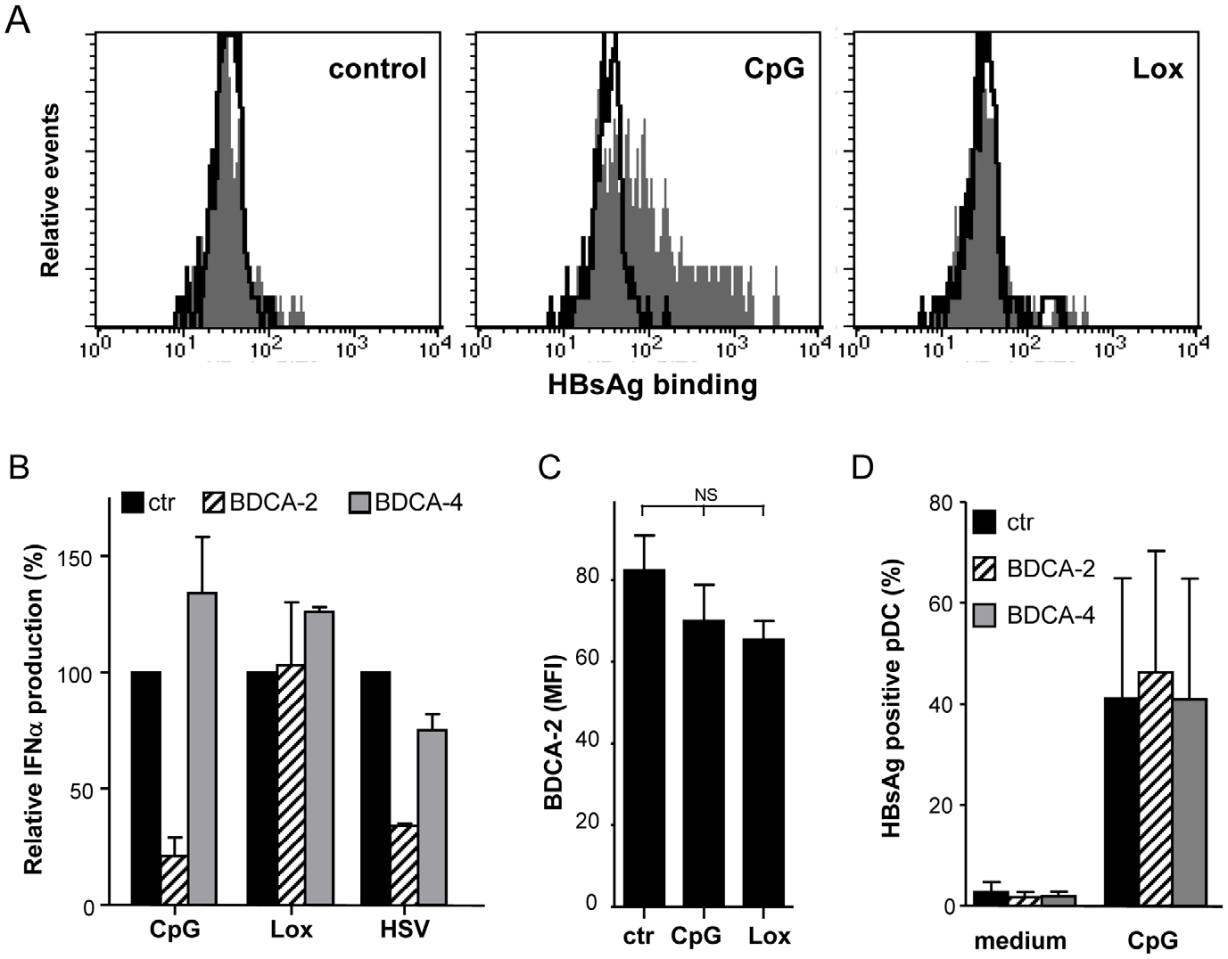
HBsAg preferentially binds to CpG-activated pDC. **A**: pDC were cultured with or without Lox or CpG and with or without HBsAg. After 4h, cells were harvested and surface bound HBsAg was detected by flow cytometry. Data are representative for 5 independent experiments. Open: HBsAg detection in cultures without HBsAg; Filled: HBsAg detection in cultures with HBsAg. **B**: PBMC were cultured in the presence of CpG, Lox or HSV-1 either with or without anti-BDCA-2 or BDCA-4. IFNα production in pDC was detected by flow cytometry. Data show mean±SD relative IFNα production compared to cultures without BDCA-2/4 crosslinking for 2 independent experiments. **C**: pDC were cultured with or without CpG or Lox for 24h. Data show mean±SEM BDCA-2 expression as assessed by flow cytometry of 5 independent experiments. NS: not significant. **D**: PBMC were cultured with or without CpG and with or without HBsAg. After 4h, cells were harvested and surface bound HBsAg was detected on pDC by flow cytometry. Data show mean±SEM HBsAg positive pDC of 5 independent experiments.

Therefore, the specific inhibition of TLR9-induced function may be more related to specific interaction of HBV and HBsAg with TLR9-stimulated pDC rather than the induction of a specific immune regulatory protein. To investigate the interaction between HBsAg, either as part of the viral envelope or single circulating protein, and pDC was examined by culturing pDC with or without CpG or Lox either in the presence or absence of HBsAg for 4h. In addition, cells were stimulated with CpG and Lox for 2h at 37°C, then cells were put on ice and HBsAg was added. Subsequently, pDC surface binding of HBsAg was determined. In both types of experiments, no or only a very low binding of HBsAg was observed in cultures with medium alone and Lox, whereas pDC stimulated with CpG strongly bound the HBV envelope protein (Fig. 8A). Hence, it is tempting to speculate that the preferential binding of HBsAg to CpG-stimulated pDC is responsible for the ability of the virus to interfere especially with CpG-induced pDC function.

Recently, BDCA-2 was proposed as receptor involved in pDC-HBsAg interaction leading to active inhibition of pDC function [14]. However, in contrast to HBV (Fig. 2A) and HBsAg (data not shown), BDCA-2 crosslinking also inhibited HSV-induced pDC function (Fig. 8B) as described previously [21]. In addition, the negligible interaction between HBsAg and non-stimulated pDC and the increased binding of HBsAg to CpG-activated pDC are in contrast to the high expression of BDCA-2 on non-stimulated cells, which is even downregulated to a limited extent upon activation (Fig. 8C) [21]. In this experimental setup, HBsAg binding was not significantly inhibited by the addition of anti-BDCA-2 antibodies (Fig. 8D). These data suggest that a CpG-induced co-factor, but not BDCA-2, is involved in the increased binding of HBsAg to pDC.

## Discussion

pDC play a central role in anti-viral immunity due to their rapid and profound release of type I IFN upon viral recognition. We demonstrated that HBV does not activate pDC as assessed by cytokine production and co-stimulatory molecule expression. Moreover, HBV interfered with TLR9-induced pDC function, resulting in dose-dependent inhibition of cytokine production and pDC maturation. HBsAg, and to a limited extent HBeAg, which were used in concentrations found in hepatitis B patients' circulation [18], showed similar immune regulatory effects as HBV thereby demonstrating that the immune regulatory effects of HBV do not require active infection.

The lack of a detectable initial type I IFN response in patients upon HBV infection is not understood so far [5], [6]. With the exception of a marginal elevated expression of HLA-DR in the presence of HBV, which may lead to enhanced DC-T cell interactions and viral immunity, the overall inability of HBV to activate pDC may underlie this defective innate immune response. Since pDC possess several pattern recognition receptors that are mainly located intracellular, the lack of pDC activation by HBV could be due to the fact that HBV is not taken up by pDC. This does not rule out the possibility that HBV indirectly activates immune receptors in/on pDC, e.g. by uptake of infected hepatocytes or crosstalk with other immune cells.

The finding that HBV-DNA could be detected in peripheral blood pDC of a subset of chronic HBV patients [15], [16] indicates direct interaction between pDC and HBV *in vivo*. The lack of pDC activation upon exposure to HBV supports the hypothesis that HBV behaves as a “stealth” virus and does not induce IFN-related genes during acute infection [5]. However, our data also support ideas that HBV possesses immunosuppressive strategies to evade the initial response that could be elicited by the innate immune system of the host. The decreased CpG-induced IFNα production by pDC exposed to HBsAg is in line with a recent study [14]. Here we showed that HBsAg not diminishes all pDC functions, but HBsAg and HBV inhibit more than only IFNα production. Not only the direct anti-viral activities, i.e. the production of cytokines, but also other important more indirect anti-viral immune parameters were influenced by HBV. pDC maturation, important for pDC-T cell crosstalk, was strongly impaired and pDC-induced NK cell function was also significantly diminished. The impaired crosstalk between different immune cells may partially explain the failing induction of effective anti-viral immunity. Whether the impaired DC-NK cell crosstalk could explain the defective IFNγ production by NK cells circulating in patients with chronic hepatitis B [22], [23] remains unknown.

Of relevance, next to HBsAg and HBV also HBeAg showed immune regulatory effects on pDC. Immunosuppressive effects of the whole virion, as well as HBsAg and HBeAg, have also been reported for TLR3 and TLR4-mediated innate immunity of murine hepatocytes and non-parenchymal liver cells [24], [25]. The lack of direct pDC activation combined with the inhibitory effect of HBV on the innate immune function of pDC, as well as other intrahepatic cells, may contribute to the enhanced risk of viral persistence upon infection with HBV compared to other viruses such as Influenza. Nevertheless, infection with HBV at adult life most often leads to self-limited acute hepatitis B. This could be explained by direct and/or indirect immune stimulatory effects of HBV towards other cells of the immune system, such as recently demonstrated for Kupffer cells [26]. It is likely that in most HBV-infected adults these immune stimulatory processes overrule the inhibitory effects of the virus leading to adequate anti-HBV specific immunity.

The inhibitory effect of HBsAg was completely overlapping with HBV, as demonstrated by inhibition of S6 and IRF7 phosphorylation and production of IFNα, TNFα, IL-6 and IP-10. HBeAg impaired pDC function to a much lesser extent and seemingly via another yet unknown intracellular signalling mechanism. At what level HBV and HBsAg interfere with mTOR-induced S6 phosphorylation remains to be elucidated. Several putative binding factors have been described for HBsAg, but their exact role in HBV attachment remains unclear [27]–[29]. The increased binding of HBsAg to CpG-stimulated pDC suggests the involvement of a TLR9-induced co-factor, either membrane bound or soluble, that scavenges HBV/HBsAg. Whether TLR9 triggering occurs in HBV-infected individuals is not known. It is tempting to speculate that pDC could be indirectly activated by HBV via interaction with viable or dead HBV-infected cells that contain viral DNA able to trigger TLR9, but also other HBV-related or even unrelated activation signals may induce the expression of this scavenging co-factor. Differential expression of this factor, e.g. related to different disease states or host polymorphisms, may influence pDC function and the regulation of virus-specific immunity.

Since HBV surface antigens are glycoproteins, the involvement of a C-type lectin seems plausible. Crosslinking of the pDC-specific C-type lectin DC immunogenic receptor (DCIR) resulted in a specific TLR9, but not TLR7-mediated inhibition of TNFα and IFNα [30], which is compatible to the immune modulatory effects observed for HBV. Nevertheless, these C-type lectins are often highly expressed on non-stimulated pDC and downregulated by TLR7 and TLR9 triggering to a similar extent as was observed for BDCA-2 [21], which is in contrast to the observed increased interaction between HBV and pDC upon CpG stimulation. Altogether, we cannot confirm the binding of HBsAg to BDCA-2 to explain the inhibitory effect of HBsAg on pDC function as suggested before [14], which indicates that there is at least also another receptor involved. Several other viruses, including HCV and HIV, have been shown to block TLR9-induced pDC function, but not TLR7-mediated activation [31]. These viruses may crosslink similar cellular/soluble receptors as HBV, but whether they also preferentially bind to TLR9/CpG-activated pDC is not documented.

In line with our *in vitro* observations that HBV significantly interferes with CpG-induced, but hardly affects Lox-induced pDC function, also chronic HBV patients displayed a significant defect in CpG-induced, but not Lox-induced IFNα production by pDC compared to healthy controls. Impaired pDC function, mainly investigated upon exposure to TLR9 ligands, has been reported in several other studies (reviewed in Woltman et al. [13]) and has recently been associated with decreased TLR9 expression levels in chronic HBV [32]. Whether decreased TLR9 expression levels explain the specific impairment in CpG-induced IFNα production as demonstrated in the present study remains to be elucidated. Previously, the loss of serum HBeAg during anti-viral treatment of patients has been suggested to be responsible for partial restoration of IFN-α production by pDC [33], [34]. In line, we here observed that especially HBeAg-positive chronic HBV patients displayed impaired pDC function compared to HBeAg-negative patients and healthy controls. These findings may be ascribed to the inhibitory effects of HBeAg on IFNα and TNFα production by pDC and at least fit with the idea that HBeAg possesses immune regulatory properties [35]. However, the HBeAg-positive patients showed higher serum ALT levels compared to the HBeAg-negative patients. Serum ALT levels inversely correlated with pDC function, which indicates that also other regulatory mechanisms than the virus itself may influence pDC function. Despite a significant inhibitory effect of HBV on pDC, both *in vitro* and *in vivo*, their function is only partially diminished and hence prevents HBV-infected individuals from generalized immune suppression. As also discussed elsewhere [13], the interaction between HBV and a subset of the total DC pool may have profound effects on the induction of specific anti-HBV immunity, but the functionality of the total DC pool and the immunocompetence of the patient seems to be retained albeit to a somewhat lower extent compared with healthy individuals.

In addition to the direct effect of HBV on pDC, HBV seems also able to indirectly influence pDC function by interfering with monocyte-pDC interaction. Whereas monocytes enhanced cytokine production by pDC under control conditions, monocytes where unable to do so in the presence of HBV. Since in contrast to HCV, we neither detected HBV-induced TNFα or IL-10 in monocytes (data not shown) nor restored HBV-induced regulation by IL-10 neutralization, it is tempting to speculate that HBV abrogates this monocyte-induced pDC function by interfering with monocyte-pDC interaction rather than active induction of an immune regulatory factor by monocytes. Of note, interaction between HBV/HBsAg and monocytes have been reported [36], but the mechanism underlying monocyte-mediated regulation of pDC function is not known.

Since a considerable number of patients chronically infected with HBV do not demonstrate an adequate anti-HBV response upon treatment with standard immune modulatory therapy, novel anti-viral strategies are needed. For the treatment of chronic HCV, promising results were obtained with the TLR9 agonist CpG10101 [37]. The finding that HBV strongly interacts with CpG-activated cells may disrupt the anti-viral effects of such immune stimulating agents. Therefore, the data presented here not only provide new insight into the mechanism by which HBV is able to evade anti-viral immunity, but they may also aid in the development of effective immunomodulatory therapies for the treatment of chronic HBV.

## Materials and Methods

### HBV, proteins and TLR ligands

HepG2.215-derived HBV particles were purified and quantified as described before [38] and unless indicated otherwise used at 100 or 2000 geq/cell for PBMC and pDC cultures, respectively. As a negative control, the same procedure was followed with supernatant from untransfected HepG2 cells. Additionally, patient-derived HBV was purified from serum of a chronic HBV patient (genotype B, viral load 3.28×10^9^ IU/ml) using the same procedure. Serum of a healthy control was treated similarly and was used as a control. Recombinant Chinese Hamster Ovary cell (CHO-)derived HBsAg and HBeAg or HBcAg derived from E. coli (Prospec, Rehovot, Israel) were used at 5µg/ml and added, like the HBV particles, either in the absence or presence of Polymyxin B (50 µg/ml; Sigma-Aldrich, St. Louis, MO). G.M.G.M. Verjans and G.F. Rimmelzwaan (both Dept of Virology, Erasmus MC) provided HSV-1 (MOI 10) and Influenza virus (H1a, MOI 0.2), respectively. Synthetic TLR ligands included CpG-2336 (10 µg/ml, Coley Pharma, Düsseldorf, Germany) and Loxoribine (Lox 0.4 mM, Invivogen, San Diego, CA). In all experiments, HBV or viral proteins were added right before the addition of synthetic TLR ligands or other pDC-stimulating agents.

### Patients and healthy subjects

Peripheral heparinized blood samples were obtained from 25 patients with chronic hepatitis B (Table 2). All patients were negative for antibodies against hepatitis C, hepatitis D and human immunodeficiency virus, and did not receive treatment at time of blood donation. A matched control group comprised 21 healthy subjects. The study was approved by the local ethics committee, and all patients and controls in the study gave informed consent before blood donation.

### Cell purification and culture

PBMC and pDC were isolated from peripheral heparinized blood samples or buffy coats from healthy blood donors using Ficoll density gradient centrifugation. All healthy controls gave written informed consent before blood donation and the institutional medical ethical committee gave declaration of no objection for this study. pDC were isolated by CD19+ cell depletion, anti-Blood Dendritic Cell Antigen (BDCA-4)-PE and anti-PE MACS microbeads (Miltenyi Biotec, Bergisch Gladbach, Germany) or FACSorting (FACS Aria, Beckton Dickinson, Alphen a/d Rijn, The Netherlands). Purity and viability (both >95%) were checked using anti-BDCA2-FITC (Miltenyi) and 7-AAD (eBioscience, San Diego, USA) by flow cytometry. Monocytes were purified with anti-CD14 MACS microbeads and MS-columns (Miltenyi). 1×10^5^ pDC, isolated by FACsorting, were co-cultured with NK cells, isolated from the same donor with an NK cell isolation kit (Miltenyi Biotec, Germany), in a 1∶5 ratio in RPMI 1640 containing 10% FCS, penicillin/streptomycin, Hepes, IL-3 and CpG either with or without HBV (200 geq/pDC) for 48h.

### Flow cytometric analysis: Surface markers and signalling molecules

pDC were stained with anti-BDCA4-PE, anti-BDCA-2-FITC, anti-CD80-FITC (MAB104; Immunotech, Marseilles, France), anti-CD123-biotin (BD Pharmingen, USA), anti-HBsAg-FITC (Acris Antibodies GmbH, Hiddenhausen, Germany), anti-CD86-APC (2331), anti-HLA-DR-PerCP (243), anti-CD40-APC (5C3) and/or streptavidin-PerCP (all BD Biosciences) in PBS/1%FCS/0.02%NaN_3_. HBsAg binding experiments were performed in the presence or absence of 5µg/ml anti-BDCA2-biotin or anti-BDCA4-PE. NK cells were stained with antibodies directed against CD56 (MY31) and CD25 (2A3, all BD Bioscience). Corresponding isotype-matched control antibodies were used to determine background staining.

To determine intracellular signalling, PBMC (1×10^6^ cells/250 µl) or pDC (5×10^3^ cells/250 µl) were stimulated at 37°C. After 5–180 min, cells were fixed with 2% formaldehyde, washed with PBS/1%FCS/0,02%NaN_3_ and incubated with 0,5% saponin. Antibodies against Phospho-S6 (pSer235/236, Bioké, Leiden, The Netherlands) and phospho-Interferon regulatory factor-7 (P-IRF7; pS477/pS479; K47-671, BD Biosciences) followed by goat-anti-rabbit-biotine (Dako, Glostrup, Denmark) were diluted in 0,5% saponin and added for 15 minutes. Finally, cells were stained with streptavidin-PerCP (BD Biosciences).

Cells were analysed by flow cytometry (FACS CantoII or FACScalibur) and FACS Diva or CellQuest Pro software (all Beckton Dickinson, Alphen a/d Rijn, The Netherlands).

### Cytokine production

PBMC (1×10^6^ cells/ml) or pDC (2×10^4^ cells/ml) were resuspended in RPMI 1640 (Lonza, Basel, Switzerland) containing 10% heat-inactivated FCS (Hyclone, Logan UT), 100U/ml Penicillin, 100µg/ml Streptomycin (Breda, The Netherlands) and IL-3 (20ng/ml, Miltenyi). Neutralizing antibodies (5µg/ml) to IL-10 or IL-10 receptor (IL-10R; both from BD Pharmingen) were added where indicated. 24h culture supernatants were examined for TNF-α (eBioscience), IL-6 (Biosource International, Nivelles, Belgium), IL-8 (Biosource), IP-10 (Invitrogen) and IFNα (Bender Medsystems, Vienna, Austria) by ELISA. IFNγ production by NK cells was also determined by ELISA (eBioScience). The frequencies of IFNα and TNFα producing pDC were quantified by incubating cells during the last 3h of 5h cultures with 10µg/ml Brefeldin A (Sigma-Aldrich). Cells were fixed and permeabilized with Intraprep (Beckman Coulter, Miami, Florida, USA) and stained with anti-IFNα-FITC (Kordia, Leiden, The Netherlands), anti-TNFα-APC (Caltag-Medsystems, Buckingham, UK) and pDC-specific antibodies as described above.

### Quantitative RT-PCR

Cells were lysed in Trizol (Sigma-Aldrich) and stored at −80°C until further use. RNA was precipitated with 75% ethanol and isolated using RNeasy mini colums (Qiagen, Venlo, The Netherlands). cDNA was synthesized with iScript cDNA synthesis kit (BioRad laboratories BV). Real-time PCR was performed with the following primer pairs: GAPDH_F 5′-AGG TCG GTG TGA ACG GAT TTG-3′ and GAPDH_R 5′TGT AGA CCA TGT AGT TGA GGT CA-3′, IFNα2_F 5′-AAT GGC CTT GAC CTT TGC TT-3′ and IFNα2_R 5′-CAG CTT GAC TTG CAG CTG AG-3′, IFNα8_F 5′-TGG TGC TCA GCT ACA AGT CAT T-3′ and IFNα8_R 5′-TAC CCA GGC TGT GAG TCT GA-3′ under standard conditions (annealing temperature 63°C; 40 cycli; MyIQ iCycler, Biorad). IFNα gene expression was normalized to GAPDH and calculated using the ΔΔCT method [39].
